# Is there any association between osteoporotic vertebral fracture and vitamin K epoxide reductase complex subunit-1 polymorphism in Turkish society? A pilot study

**DOI:** 10.6061/clinics/2019/e739

**Published:** 2019-03-05

**Authors:** Merih Ozgen, Didem Turgut Cosan, Fulya Doganer, Ahu Soyocak, Onur Armagan, Selen Kuzgun, Ayse Merve Aydogan, Hasan Veysi Gunes, Irfan Degirmenci, Fezan Mutlu

**Affiliations:** I Department of Physical Medicine and Rehabilitation, Faculty of Medicine, Eskisehir Osmangazi University, Eskisehir, Turkey; II Department of Medical Biology, Faculty of Medicine, Eskisehir Osmangazi University, Eskisehir, Turkey; III Department of Biotechnology and Molecular Biology, Faculty of Arts and Sciences, Aksaray University, Aksaray, Turkey; IV Department of Medical Biology and Genetics, Faculty of Medicine, Istanbul Aydin University, Istanbul, Turkey; V Department of Physical Medicine and Rehabilitation, Zübeyde Hanım Campus, Eskişehir State Hospital, Eskisehir, Turkey; VI Department of Medical Biology, Faculty of Medicine, Kutahya Health Sciences University, Kutahya, Turkey; VII Department of Biostatistics and Medical Informatics, Faculty of Medicine, Eskisehir Osmangazi University, Eskisehir, Turkey

**Keywords:** Osteoporosis, Polymorphism, Vitamin K Epoxide Reductase Complex Subunit-1 Gene, Vertebral Fracture

## Abstract

**OBJECTIVE:**

In this study, the relationship between osteoporotic vertebral fractures and 9041 Guanine/Adenine and 3673 Guanine/Adenine polymorphisms related to the vitamin K epoxide reductase complex subunit-1 (VKORC1) gene in postmenopausal women with osteoporosis was investigated.

**METHOD:**

DNA was isolated from blood samples collected from 150 women with postmenopausal osteoporosis. Genotyping of the two polymorphic regions (9041 Guanine/Adenine and 3673 Guanine/Adenine) in VKORC1 was performed using polymerase chain reaction–restriction fragment length polymorphism analysis. The presence of radiographic fractures among the 150 patients was ascertained by using the Genant method.

**RESULT:**

At least one fracture was detected in 98 patients, and no fracture was observed in 52 patients on radiological images. We found no association between the 9041 Guanine/Adenine (*p*=0.283) and 3673 Guanine/Adenine (*p*=0.232) polymorphisms of the VKORC1 gene and the development of secondary postosteoporotic fractures in our study.

**CONCLUSION:**

There was no relationship between osteoporotic vertebral fracture and VKORC1 gene polymorphism in a postmenopausal Turkish population.

## INTRODUCTION

Osteoporosis is a disease that causes general health problems associated with low bone mass and microarchitectural damage in bone tissue. These changes in bone structure cause increased bone fragility and fracture susceptibility 1. The increased prevalence of osteoporosis-associated fractures with increased life span has resulted in lower quality of life, disability and even death 2. Environmental factors such as nutritional and genetic factors play an important role in osteoporosis 3,4.

According to recent studies, vitamin K could be associated with bone metabolism and may provide protection against fractures 5-8. Vitamin K is a co-factor that involves many biochemical pathways related to carboxylation reactions 9. The carboxylation of osteocalcin, which is an important bone matrix protein, depends on vitamin K. Vitamin K epoxide reductase complex subunit 1 (VKORC1) enzyme activity is necessary for the effect of vitamin K on bone metabolism 9-11. In humans, the VKORC1 gene is found in the 16p11.2 region of the chromosome, coding a 163-amino acid protein with a molecular weight of 18 kDa 12. In the VKORC1 gene, many single nucleotide polymorphisms (SNPs) have been identified, including SNPs 1173 (6484) C/T (rs9934438) in intron 1, 9041 (3730) Guanine (G)/Adenine (A) (rs7294) in the 3′-untranslated region and 3673 (1639) G/A (rs9923231) in the promoter 13.

Many reports on vitamin K treatment in osteoporosis and related fractures have been published 7,14-16. However, there have been few studies on the association between osteoporosis and polymorphisms of the VKORC1 enzyme 11,17. In addition, in the literature, there are no studies on the relationship between genetic variations in the VKORC1 gene and bone mineral density (BMD) and osteoporotic fractures in the Turkish population.

Determination of the association of some vitamin K-related polymorphisms with osteoporosis and osteoporosis fractures could be used for clinical prevention and the planning of individual treatment targets. Therefore, we aimed to investigate VKORC1 9041 G/A and 3673 G/A polymorphisms that varied among populations in osteoporosis and postosteoporotic vertebral fractures in Turkish women with postmenopausal osteoporosis.

## MATERIALS AND METHODS

### Subjects

This study included 150 female patients with postmenopausal osteoporosis.

### Inclusion Criteria

Thoracal and lumbar lateral radiographs and double energetic X-ray absorptiometry (DXA) results of female patients with postmenopausal osteoporosis were retrospectively analyzed in Physical Therapy and Rehabilitation clinics of Eskisehir Osmangazi University. Patients with normal complete blood count, calcium, phosphorus, erythrocyte sedimentation rate, serum alkaline phosphatase, vitamin D, glucose, aspartate aminotransferase, albumin, alanine aminotransferase, gamma-glutamyl transpeptidase, uric acid, urea nitrogen, blood creatinine, urine calcium creatinine ratio, parathyroid hormone, thyroid-stimulating hormone and cortisol levels were included in this study.

### Exclusion Criteria

Patients with a history of traumatic fractures, surgical menopause, or medical conditions that have a potential effect on bone metabolism (e.g., parathyroid, thyroid, liver or rheumatic disease or malignancy) or drug use (corticosteroids, anticonvulsants, heparin, etc.) were excluded from the clinical evaluation.

At least one fracture was detected in 98 patients, and no fracture was observed in 52 patients on radiological images. Body mass index (BMI) and age were recorded. The working procedures have been approved in writing by Eskişehir Osmangazi University Faculty of Medicine Ethics Committee (no. 2009/229). The DNA samples collected for the study were used in accordance with the decision of the ethics committee no. 2012/103.

### Bone Mineral Density (BMD) Measurement

DXA (Hologic QDR, 4500 W) was used to measure bone density in the lumbar vertebral and femoral bone (femur neck, trochanter and Ward triangle). Bone density test results were evaluated as T-scores and Z-scores. According to WHO recommendations, patients with a T-score below -2.5 of the young adult mean value standard deviation were classified as osteoporosis 18.

### Radiographic Assessment

The presence of radiographic fractures among the 150 patients was ascertained by using the Genant method. In this method, the rate of the decrease in the middle and/or posterior height of T4-L4 vertebrae in the lateral spinal X-rays (Shimadzu, RADspeed PRO, MO2ABC775003/TAEK13136, Japan) was used. Fracture presence was indicated by loss of height in at least one vertebra between T4 and L4 in lateral spinal radiography 19.

### Sample Collection

Blood samples (10 mL) were obtained from all subjects for genomic DNA extraction using the salt extraction method 20. The collected genomic DNA samples were kept at 4°C. The purity and amount of isolated DNA samples were measured by spectrophotometry (ASP 3700, ACTGene, Piscataway, NJ, USA).

### Determination of VKORC1 Genotypes

9041 G/A and 3673 G/A VKORC1 polymorphisms were determined using polymerase chain reaction–restriction fragment length polymorphism (PCR–RFLP) analysis. Briefly, isolated DNA samples were amplified using Taq DNA polymerase (Thermo Scientific, California, USA) and the specific primers for the VKORC1 polymorphisms (Table 1) in polymerase chain reaction (PCR) (Sacem Life Technologies, Peltier based Thermal Cycler SCM 96G, Turkey). The PCR conditions are shown in Table 1. Approximately 0.5 μl of DNA sample was amplified using a 25 μl PCR mixture containing 2.5 pmol from each primer, 1x PCR buffer with magnesium chloride (New England BioLabs, Ipswich, United Kingdom), 0.2 mM dNTPs, and 0.5 U Taq polymerase (New England BioLabs, Ipswich, United Kingdom). Following amplification, PCR products were digested by restriction enzymes. The 674-bp VKORC1 9041 G/A fragment was digested with AciI (New England BioLabs, Ipswich, United Kingdom) in a 25 μl reaction containing 10 μl PCR fragment, 2.5 μl Reaction Buffer (NEBuffer 3) (New England BioLabs, Ipswich, United Kingdom), 12 μl PCR grade water and 0.5 μl AciI at 37°C for 1 h. The 636-bp VKORC1 3673 G/A fragment was digested with NciI (New England BioLabs, Ipswich, United Kingdom) in a 25 μl reaction containing 10 μl PCR fragment, 2.5 μl Reaction Buffer (NEBuffer 4) (New England BioLabs, Ipswich, United Kingdom), 12.4 μl PCR grade water and 0.1 μl of NciI at 37°C for 1h. The digested products were analyzed for the presence or absence of recognition sites by SYBR^®^ Safe DNA gel stain (Invitrogen, Paisley, United Kingdom) of fragments separated through a 2% agarose gel and analyzed under UV light (Synegene Gene Genius Gel Light Imaging System, Cambridge, United Kingdom). Digested patterns belonging to the 674 bp amplicon of 9041 G/A SNP and the 636 bp amplicon of the VKORC1 3673 G/A SNP are shown in Table 1.

### Statistical Analysis

Statistical analyses were calculated by using IBM SPSS Statistics 21.0 (SPSS Inc., Chicago, IL, USA). Descriptive statistics are displayed as the mean ± standard deviation (SD) and median (%25, %75) for continuous variables and number and percentage with characteristics for categorical variables. Pearson chi-square analysis was used for categorical variables. Multiple logistic regression analysis was performed for 9041 G/A and 3673 G/A in postmenopausal osteoporosis patients with and without fractures. The results of analysis, odds ratio (OR) and a 95% confidence interval (CI) are displayed. All *p*-values <0.05 were accepted as statistically significant.

## RESULTS

A total of 150 female patients with postmenopausal osteoporosis were included in the study to investigate the relationship between postosteoporotic spinal fractures and the distribution of 9041 G/A and 3673 G/A polymorphisms in the VKORC1 gene. In our study, the mean age of women with postmenopausal osteoporosis was 61.78±8.11, the mean BMI was 27.78±4.19, the mean T score in the femoral neck was -2.93±0.41, and the mean T score in the lumber vertebrae was -2.83±0.75. Radiographic assessments showed the presence of at least one fracture in 98 patients with osteoporosis.

There was no relationship between the VKORC1 9041 G/A polymorphism and allele frequency in patients with and without postmenopausal osteoporosis (*p*=0.283, *p*=0.221). However, the mutant A allele frequency was decreased in 67 (34.18%) postmenopausal women with osteoporosis and fractures compared with that in 129 (41.35%) patients with no fractures (Table 2). In patients with vertebral fractures compared with those with no fractures**,** the frequency of the AA allele was 1.73 times smaller than that of the GG allele. However, this increase was not statistically significant (OR=0.568, 95% CI 0.276, 1.158 *p*=0.120) (Table 3).

Similar to the 3673 G/A polymorphism and allele frequency, the genotype distribution of this polymorphism also did not differ between patients with osteoporosis with or without fractures (*p*=0.232, *p*=0.851). However, the mutant A allele frequency was decreased in 104 (53.06%) postmenopausal women with osteoporosis with fractures compared with that in 92 (51.92%) patients with no fractures (Table 2). In patients with vertebral fractures compared with patients with no fractures, the frequency of the AA allele was 1.535 times higher than that of the GG allele. However, this increase was nonsignificant (OR=1.533, 95% CI 0.723, 0.271, *p*=0.266) (Table 3).

## DISCUSSION

The genetic factors of osteoporosis are important for understanding the diagnosis and development of new treatment methods. In studies that investigated the genetic basis of osteoporosis and osteoporotic fractures, several candidate genome-wide association studies (GWAS) have been performed, and SNPs, which may be associated with the development of osteoporosis, have been identified 21-25.

Based on previous studies, the VKORC1 haplotype profile varies between large populations of different origins 26. Moreover, considering that BMD distribution varies per population, genetic variants of the VKORC1 gene may be associated with BMD and osteoporosis. The 3673 G/A and 1173 C/T SNPs of the VKORC1 SNP genotype have been associated with higher BMD values, while polymorphisms in VKORC1 (rs8050894 and rs2884737) are related to reduced BMD. Furthermore, the association between VKORC1 and BMD reportedly exhibits certain variations according to populations 17. In studies that have investigated the association between BMD, atherosclerosis and VKORC1 1173 C>T polymorphisms in patients with osteoporosis or osteopenia, a high frequency of the TT genotype has been found in VKORC1 1173 C>T polymorphisms. The TT genotype is more common in the osteoporotic group than in the osteopenic group, and the TT genotype of VKORC1 1173 C>T could be a potential genetic marker for osteoporosis 27. The effect of VKORC1 polymorphisms on BMD and fractures was analyzed in a pilot study. In that study, genotyping for VKORC1 3673 G/A or 9041 G/A derivatives was performed using allele-specific PCR in 149 of the 184 individuals, and an association was observed between genotypes and clinical parameters. Significantly high levels of 9041 GG and GA were detected in patients with low BMD (*p*=0.012). Therefore, a higher risk of low BMD was identified for individuals carrying at least 1 G allele. No significant correlation was found between the 3673 G/A derivative and BMD, and none of the variants were associated with fractures. In this study, genetic variation in the 3′ locations of the VKORC1 gene (9041 AG and GG) was correlated with significantly reduced BMD. Therefore, genetic variations may have an important role in osteoporosis. In our study, the association of the VKORC1 gene polymorphisms with both osteoporosis and osteoporotic fractures was examined. In our study, similar to the study by Holzer et al. 11, the relationship between the development of osteoporosis and the 9073 G/A and 3673 G/A polymorphisms of the VKORC1 gene were investigated, and no correlation was found between these polymorphisms. In addition, there was no correlation between 9073 G/A and 3673 G/A polymorphisms and vertebral fractures associated with postmenopausal osteoporosis in our study.

Our study has several limitations that must be considered. The number of patients in our study was small in terms of statistical power analysis. There were no data on the distribution of VKORC1 9041 G/A and 3673 G genotypes in a Turkish population. In addition, there was insufficient information on the nutritional patterns and blood vitamin K levels that may be associated with osteoporotic vertebral fractures in female patients with postmenopausal osteoporosis. To our best knowledge, this study is the first to investigate the relation between VKORC1 genotypes and vertebral fracture among postmenopausal women in the literature and Turkish society.

## CONCLUSION

Genetic studies with patients with osteoporosis may allow the development of new strategies for the widely available therapeutic agents or the identification of target molecules for new therapeutic agents. The present study was planned to demonstrate a possible relationship between vertebral fracture and polymorphisms of the VKORC1 gene in osteoporosis. As a result of this study, there was no relationship between osteoporotic vertebral fracture and VKORC1 gene polymorphism in a postmenopausal Turkish population. According to the results of the logistic regression analysis of our study, if the number of patients was increased, there may be a relationship in terms of alleles. For this reason, there is a need for multicenter studies with wider populations, including all osteoporosis subgroups.

## AUTHOR CONTRIBUTIONS

Ozgen M and Cosan DT conceived the research, interpreted the collected data, reviewed the manuscript and approved the final version of the manuscript. Doganer F, Soyocak A, Kuzgun S and Degirmenci I collected the data, helped in interpreting the collected data and approved the final version of the manuscript. Armagan O conceived the research, reviewed the manuscript and approved the final version of the manuscript. Aydogan AM and Gunes HV reviewed the manuscript and approved the final version of the manuscript. Mutlu F interpreted the collected data, reviewed the manuscript and approved the final version of the manuscript.

## Figures and Tables

**Table 1:** PCR protocols and primer sequences for amplification of different loci of the vitamin K reductase complex subunit 1 (VKORC1) gene.

Loci	Primers		Size and fragments		PCR protocol
9041 G/A	F-5′-TTTAGAGACCCTTCCCAGCA -3′R-5′-AGCTCCAGAGAAGGCAACAC-3′	674 bp	GG	117 bp 216 bp 341 bp	95°C for 30 s 59°C for 1 min 72°C for 1 min for 35 cyc
			GA	117 bp 216 bp 341 bp 557 bp	
			AA	117 bp 557 bp	
3673 G/A	F- 5′-ATCCCTCTGGGAAGTCAAGC-3′R- 5′-CACCTTCAA CCTCTCCATCC-3′	636 bp	GG	50 bp 114 bp 472 bp	95°C for 30 s 60°C for 1 min 72°C for 1 min for 35 cyc
			GA	50 bp 114 bp 472 bp 522 bp	
			AA	114 bp 522 bp	

*A* Adenine, *G* Guanine, *C* Cytosine, *T* Thymine.

**Table 2:** 9041 G/A and 3673 G/A genotype and allele frequency distribution in postmenopausal osteoporosis patients with and without fracture.

Genotype		Postmenopausal Patients with Osteoporosis		*p* values
		Without Fracture (n=52)	With Fracture (n=98)	
9041 G/A	GG	16 (31%)	43 (44%)	0.283
	GA	29 (56%)	43 (44%)	
	AA	7 (13%)	12 (12%)	
Allele frequency	G	61 (58.6%)	129 (41.35%)	0.221
	A	43 (41.35%)	67 (34.18%)	
3673 G/A	GG	16 (30.8%)	22 (22.4%)	0.232
	GA	18 (34.6%)	48 (49%)	
	AA	18 (34.6%)	28 (28.6%)	
Allele frequency	G	50 (48.08%)	92 (96.94%)	0.851
	A	54 (51.92%)	104 (53.06%)	

*A* Adenine, *G* Guanine, *C* Cytosine, *T* Thymine.

**Table 3:** 9041 G/A and 3673 G/A in postmenopausal patients with osteoporosis with and without fracture.

Genotype		OR (95% Cl)	*p* values
9041 G/A	GG	1 (reference)	0.120
	AA+GA	0.568 (0.279-1.158)	
3673 G/A	GG	1 (reference)	0.266
	AA+GA	1.535 (0.721-3.271)	

*A* Adenine, *G* Guanine, *C* Cytosine, *T* Thymine.
